# Increasing the quantity and quality of searching for current best evidence to answer clinical questions: protocol and intervention design of the MacPLUS FS Factorial Randomized Controlled Trials

**DOI:** 10.1186/s13012-014-0125-9

**Published:** 2014-09-20

**Authors:** Thomas Agoritsas, Emma Iserman, Nicholas Hobson, Natasha Cohen, Adam Cohen, Pavel S Roshanov, Miguel Perez, Chris Cotoi, Rick Parrish, Eleanor Pullenayegum, Nancy L Wilczynski, Alfonso Iorio, R Brian Haynes

**Affiliations:** Health Information Research Unit (HiRU), CRL Building, #135, Department of Clinical Epidemiology and Biostatistics, McMaster University, Faculty of Health Sciences, 1280 Main Street West, Hamilton, L8S 4 K1 ON Canada; Department of Surgery, McMaster University, Hamilton, ON Canada; Adam Cohen Web Designs, Hamilton, ON Canada; Schulich School of Medicine and Dentistry, Western University, London, ON Canada; Formerly with the HiRU, Department of Clinical Epidemiology and Biostatistics, McMaster University, Faculty of Health Sciences, Hamilton, ON Canada; Child Health Evaluative Sciences, Hospital for Sick Children, Toronto; Dalla Lana School of Public Health, University of Toronto, Toronto, ON Canada

**Keywords:** Evidence-based medicine, Evidence retrieval, Knowledge translation, Audit and feedback, Web-based resources, Search engines

## Abstract

**Background & aims:**

Finding current best evidence for clinical decisions remains challenging. With 3,000 new studies published every day, no single evidence-based resource provides all answers or is sufficiently updated. McMaster Premium LiteratUre Service - Federated Search (MacPLUS FS) addresses this issue by looking in multiple high quality resources simultaneously and displaying results in a one-page pyramid with the most clinically useful at the top. Yet, additional logistical and educational barriers need to be addressed to enhance point-of-care evidence retrieval. This trial seeks to test three innovative interventions, among clinicians registered to MacPLUS FS, to increase the quantity and quality of searching for current best evidence to answer clinical questions.

**Methods & design:**

In a user-centered approach, we designed three interventions embedded in MacPLUS FS: (A) a web-based Clinical Question Recorder; (B) an Evidence Retrieval Coach composed of eight short educational videos; (C) an Audit, Feedback and Gamification approach to evidence retrieval, based on the allocation of `badges’ and `reputation scores.’

We will conduct a randomized factorial controlled trial among all the 904 eligible medical doctors currently registered to MacPLUS FS at the hospitals affiliated with McMaster University, Canada. Postgraduate trainees (n = 429) and clinical faculty/staff (n = 475) will be randomized to each of the three following interventions in a factorial design (A x B x C). Utilization will be continuously recorded through clinicians’ accounts that track logins and usage, down to the level of individual keystrokes. The primary outcome is the rate of searches per month per user during the six months of follow-up. Secondary outcomes, measured through the validated Impact Assessment Method questionnaire, include: utility of answers found (meeting clinicians’ information needs), use (application in practice), and perceived usefulness on patient outcomes.

**Discussion:**

Built on effective models for the point-of-care teaching, these interventions approach evidence retrieval as a clinical skill. If effective, they may offer the opportunity to enhance it for a large audience, at low cost, providing better access to relevant evidence across many top EBM resources in parallel.

**Trial registration:**

ClinicalTrials.Gov NCT02038439.

**Electronic supplementary material:**

The online version of this article (doi:10.1186/s13012-014-0125-9) contains supplementary material, which is available to authorized users.

## Background

Translation of new knowledge from research into evidence-informed health care is a shared obligation of the clinical and the scientific communities. Unfortunately, studies investigating quality of care continue to show that this goal is substantially unrealized. Clinicians’ uptake of validated best care procedures remains stubbornly around 50% or less for most advances in therapeutics [1],[2]. Combined with a similar rate of patient adherence with self-administered treatments [3], the average effectiveness of therapies reaches typically only about a quarter (50% - 50%) of their potential.

One main barrier to achieving evidence-based care by clinicians is lack of quick and easy identification, appraisal and synthesis of current best evidence. Clinicians’ information needs are considerable - with an average of five to eight questions about individual patients per daily shift [4]-[6], thus making evidence retrieval an essential skill in clinical practice [7]. However, about 3,000 articles are published in Medline every day [8], including 75 randomized controlled trials and 11 systematic reviews [9]. Numerous Evidence-Based Medicine (EBM) resources have been developed to filter and disseminate the evidence. But although increasingly used by clinicians [10]-[12], each resource offers a fragmented and scattered view of the information, and none provides comprehensive topic coverage [13],[14] or consistent and satisfactory updating [15],[16]. As a result, up to 64% of clinical questions remain unanswered, and many answers are not based on current best evidence [17]-[19].

**Figure 1:**
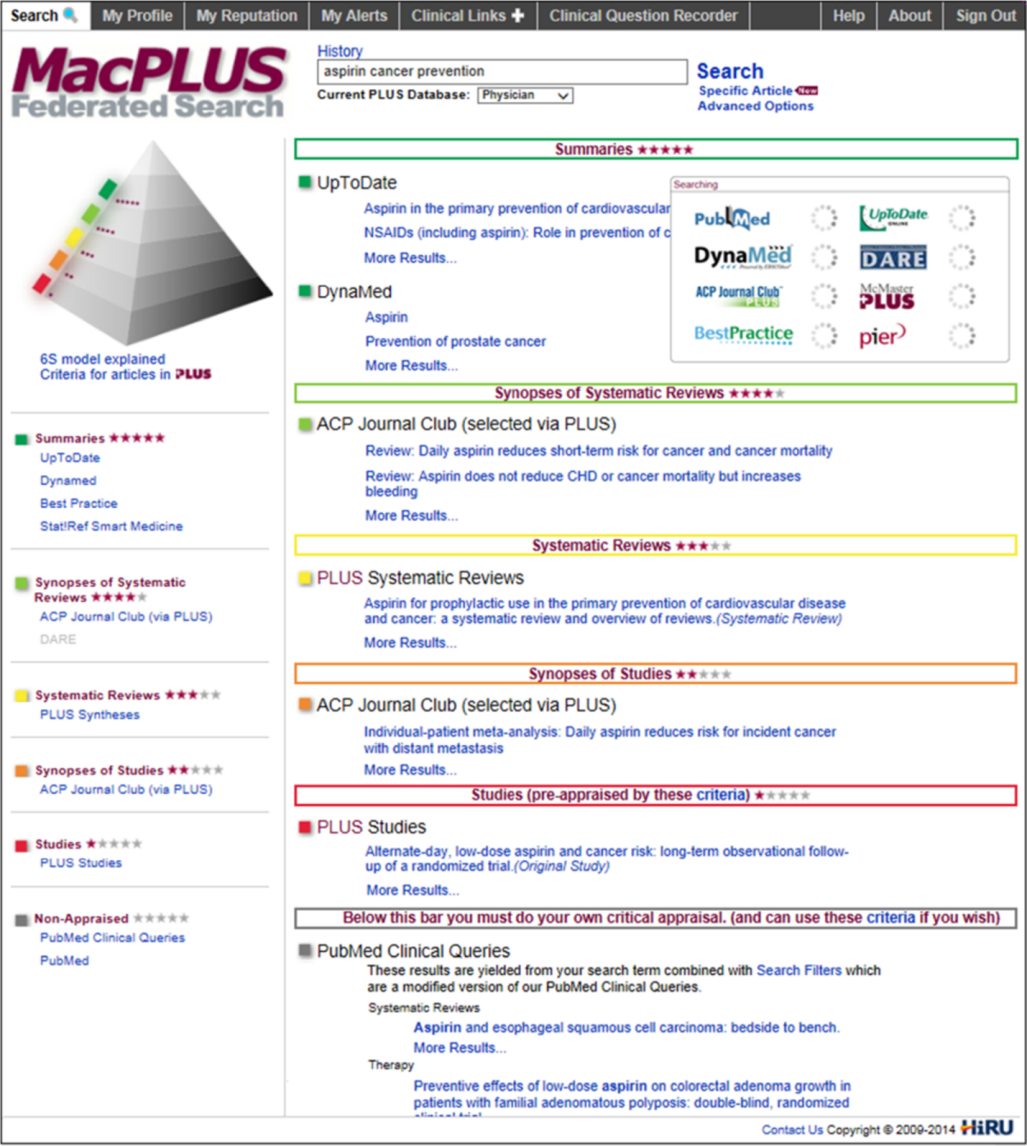
**MacPLUS FS search output.**

To address these problems, the McMaster’s University Health Information Research Unit has developed and implemented the MacPLUS Federated Search (MacPLUS FS). This novel resource provides a unique one-stop simultaneous search of multiple current best EBM resources for use at the point of care (see Table 1). It also organizes information according to the `pyramid of EBM resources,’ displaying results in one-page output with the most clinically useful at the top [20] (see Figure 1). Thus, MacPLUS FS simultaneously retrieves evidence from online summaries in the top layers (*e.g*., DynaMed, UpToDate, Best Practice, ACP Smart Medicine), then pre-appraised research in the middle layers (*i.e*., Systematic reviews, Studies and their Synopses when available, selected in McMaster PLUS database for methodological rigor and clinical relevance [21]), and finally non-pre-appraised research in the bottom layers, both filtered [22] and unfiltered from PubMed. In addition to the federated search, MacPLUS FS provides users with alerts to new research in their chosen disciplines [23] (similar content to the widely accessed BMJ EvidenceUpdates [24]), as well as numerous clinical and EBM practical links (see Table 1). Structurally, MacPLUS FS supplies evidence from research that is relevant to the clinical needs of students, postgrads, and independent practitioners.

**Table 1:** **EBM Resources accessible through MacPLUS Federated Search (MacPLUS FS)**

	Description	Specific resources available**
Summaries*	Summary of the body of evidence at a topic-level (not just a research question). Regularly updated (variable frequency).	DynaMed
		UpToDate
		Best Practice
	May provide actionable recommendations.	ACP PIER
Pre-appraised research*	Continuously updated and appraised.	
Synopses of systematic	One-page description of selected reviews with commentaries from experts.	ACP Journal Club (selected via PLUS), Database of Abstracts of Reviews of Effects (DARE)
reviews		
Systematic reviews	Selected reviews rated by clinicians	McMaster PLUS (including Cochrane)
	for relevance & novelty.	
Synopses of studies	One-page description of selected	ACP Journal Club (selected via PLUS)
	studies with commentaries from experts.	
Studies	Selected studies rated by clinicians	McMaster PLUS
	for relevance & novelty.	
Non-pre-appraised research*	Always requires independent own appraisal.	
Filtered studies	Selection of studies using empirically	Clinical Queries in PubMed
	derived methodological filters.	
Unfiltered studies	Unselected studies from large databases.	PubMed (MEDLINE)
**Alerts to new evidence updates**	Email alerts to new evidence.	McMaster PLUS
	Customized to areas of interest.	(same as BMJ EvidenceUpdates)
**Additional resources**	Available alongside the search functions.	
Single citation matcher	Helps finding specific citations.	PubMed matcher and McMasterPLUS
Clinical vital links	Prescribing information.	Compendium of Pharmaceuticals
	Patient information.	MedlinePlus
	Medical calculators and tool sets.	MedCalc3000
Other EBM links		EBM Toolbox (Oxford Centre for EBM)
	Guidance for EBM practice.	JAMAevidence (McGraw-Hill)
	Toolboxes & appraisal spreadsheets.	Centre for EBM (Univ. Health Network) Bandolier

*These layers, adapted from the 6-S pyramid of EBM resources [20],[49], are searched simultaneously in MacPLUS FS. Results are displayed on one page output in that order, *i.e*., with the most clinically useful hits at the top (see Figure 1).

**Broad full-text access at all McMaster affiliated clinical institutions participating in the trials is provided on-site through McMaster University or Hamilton Health Sciences institutional licenses. Remote access is allowed through VPN (except for UpToDate), or depends on each user’s individual subscriptions. Searching features remain always free, as well as access to all McMaster PLUS and to any open-access content.

However, combining features of the current best EBM resources is not enough to increase prompt and reasonable use of current best evidence, as shown by the relatively low utilization of searching features by the 2,800 clinicians registered with MacPLUS FS, in contrast with their high utilization of the alerting system. Additional well-known barriers that need to be overcome include logistical barriers (time constraints, forgotten questions, and simplicity of using one’s single preferred, albeit limited, resource), as well as educational barriers (*e.g*., lack of awareness of the `architecture’ of evidence and limits of non-federated single resources, lack of knowledge and experience of what federated searches can offer, limited searching skills, and lack of reference standards among peers for finding best evidence) [19],[25]-[29].

### Study aims

The trials described in this paper seek to test three innovative interventions among clinicians registered to MacPLUS FS to overcome these logistical and educational barriers and thus potentially increase the quantity and quality of searching for current best evidence to answer clinical questions.

We have designed these interventions based on effective models for the teaching of clinical skills at the point of care, to facilitate using the search engine as a clinical tool, presenting evidence retrieval skills as true clinical skills. Results from these trials may thus provide insight into whether finding current best evidence can be learned and enhanced for a large audience of clinicians through online search engines.

## Methods

### I. Overview of study design

We plan to conduct two separate factorial randomized control trials among medical doctors registered in MacPLUS FS, one among the postgraduate trainees and one among the faculty members. Participants will be randomized to the three following web-based interventions, all linked to MacPLUS FS, in a factorial design (A x B x C):

1. Intervention A - Clinical Question Recorder, linked to MacPLUS FS
2. Intervention B - Evidence Retrieval Coach, embedded in MacPLUS FS
3. Intervention C - Audit, Feedback and Gamification on searching behaviors in MacPLUS FS

Thus, half our sample will be exposed to each intervention, all possible permutations resulting in eight distinct groups of registrants receiving or not each intervention (see Table 2). Postgraduate and faculty MDs will be randomized in two separate trials. The primary outcome of interest is utilization of MacPLUS FS, namely the number of searches/month/user to answer their questions. This primary outcome will be continuously recorded from automatic monitoring of MacPLUS FS use. Secondary questions include measures of utility (satisfaction in meeting users’ information needs), use (application of evidence in practice), and perceived usefulness in patient care and outcomes, as well as changes in the pattern of use of specific resources according to the EBM pyramid (frequency and time trends in utilization).

**Table 2:** **Factorial randomization scheme of the three interventions**

Interventions*			
A	B	C	Random group allocation
*Clinical Question Recorder*	*Evidence Retrieval Coach*	*Audit, Feedback & Gamification*	
**1**	**1**	**1**	Group 1
**1**	**1**	0	Group 2
**1**	0	**1**	Group 3
**1**	0	0	Group 4
0	**1**	**1**	Group 5
0	**1**	0	Group 6
0	0	**1**	Group 7
0	0	0	Group 8

*For each intervention, half of the sample is randomized to receiving the intervention [1] and the other half to not receiving it [0]. All factorial combinations of the intervention result in eight allocation groups (2^3^ = 8).

In the next section, we describe the development of the three interventions: our theoretical framework; user-testing of their different iterations; and the final features that we will test in the trials. The third section details the methodology of the factorial randomized controlled trials.

### II. Development of the interventions

#### Theoretical framework

To overcome the aforementioned logistical and educational barriers to answering clinical questions with current best evidence [19],[25]-[29], we have built the general framework for our three interventions on effective models for teaching clinical skills at the point of care. We have opted for that approach so that clinicians are facilitated in perceiving evidence retrieval skills as true clinical skills, and encouraged to use MacPLUS FS as the most comprehensive clinical tool for evidence retrieval, in terms of topic coverage, optimal updating, signal to noise ratio and time-management.

Many models have been developed to teach clinical skills at the point of care, but one that has been consistently shown as effective in randomized control trials, and then most widely adopted by clinical teachers, is the `One-minute preceptor model,’ also known as the `5-step Microskills’ [30]-[34]. As shown in Table 3, we have adapted the teaching steps of this model for the purpose of enhancing evidence retrieval as follows: identifying searching opportunities; prompting searches to answer clinical questions; providing general knowledge, skills and feedback; and inviting reflective practice. We have developed our three interventions (A, B & C) to map these teaching steps.

**Table 3:** **Correspondence between the one-minute preceptor model, and the interventions developed for the MacPLUS FS trial**

	One-minute preceptor teaching “steps”	Corresponding facilitators for evidence retrieval in MPFS trial	Interventions in the trial
1	Identify teaching opportunities	Identify searching opportunities by recording clinical questions.	**A**
			*Clinical Question Recorder*
2	Get a commitment	Prompt search by helping recall unanswered clinical questions.	**A**
			*Clinical Question Reminder*
3	Probe for evidence supporting clinical practice	Facilitate appropriate use of pyramid of EBM resources through continuous guidance.	**B**
			*Evidence Retrieval Coach*
4	Teach general rules	Provide tailored short videos of `small bites’ of teaching & tips on evidence retrieval.	**B**
			*Evidence Retrieval Coach*
5	Feedback (Reinforce what was done right/Correct mistakes)	Provide feedback on frequency of searches and depth of use, compared to peers. Engage with gamification.	**C**
			*Audit, Feedback & Gamification*
6	Identify next objectives	Keep track of questions answered in a virtual logbook.	**A**
	Reflective practice		*Clinical Question Recorder*

### Intervention A - clinical question recorder

#### Development methods

The purpose of this web-based intervention is to allow clinicians to: i) easily record their questions at the point of care; ii) receive periodic reminders of unanswered questions, thus providing asynchronous opportunity for evidence retrieval [35]; and iii) keep track of their questions and evidence-based answers in a virtual logbook to enhance their reflective practice. To achieve these objectives, we designed initial mock-ups and a web-based prototype of the recorder, to be linked to the clinician’s individual MacPLUS FS account and accessible across a wide range of devices (primarily smartphones for point of care use, but also tablets and computer desktops).

This intervention requires the active participation of clinicians. To maximize the likelihood that they engage, we focused our development on a user-centered approach based on iterative user-testing of sequential prototypes [36],[37]. We recruited independent testers, gave them access to the prototype on their smartphone, and exposed them to nine real-life scenarios that evaluate different aspects of the intervention during one-hour `think out loud’ sessions. Using a standardized interview guide (see Additional file 1), we observed and collected their user experience based on Peter Morville’s honeycomb framework [38]. We thus identified major and minor problems and suggestions for improvements on the following dimensions: findability, accessibility, usability, understandability, usefulness, credibility, desirability, and identification. Based on that feedback, we refined the prototype after every two to three user-tests until the problems were overcome and the intervention was intuitive and satisfactory for the users. We then implemented it on the MacPLUS FS interface, with a final check of online usability by the same users accessing it remotely from their setting.

#### User-testing

We recruited eight independent testers (three practicing MDs, one student MD, three master’s students in Health Research Methodology and one medical librarian), who underwent 12 full user-tests. We also performed numerous shorter usability tests on four team members. This process identified 34 significant issues - mainly around accessibility, usability, understandability, usefulness, and desirability - which resulted in 38 modifications of the prototype, across 5 major iterations (4 to 11 issues and 3 to 13 changes made per iterations). Consistently fewer refinements were necessary as use of the recorder became more intuitive and users were more satisfied. Final remote usability testing did not identify any remaining issues.

### Results: description of the final features

**Figure 2:**
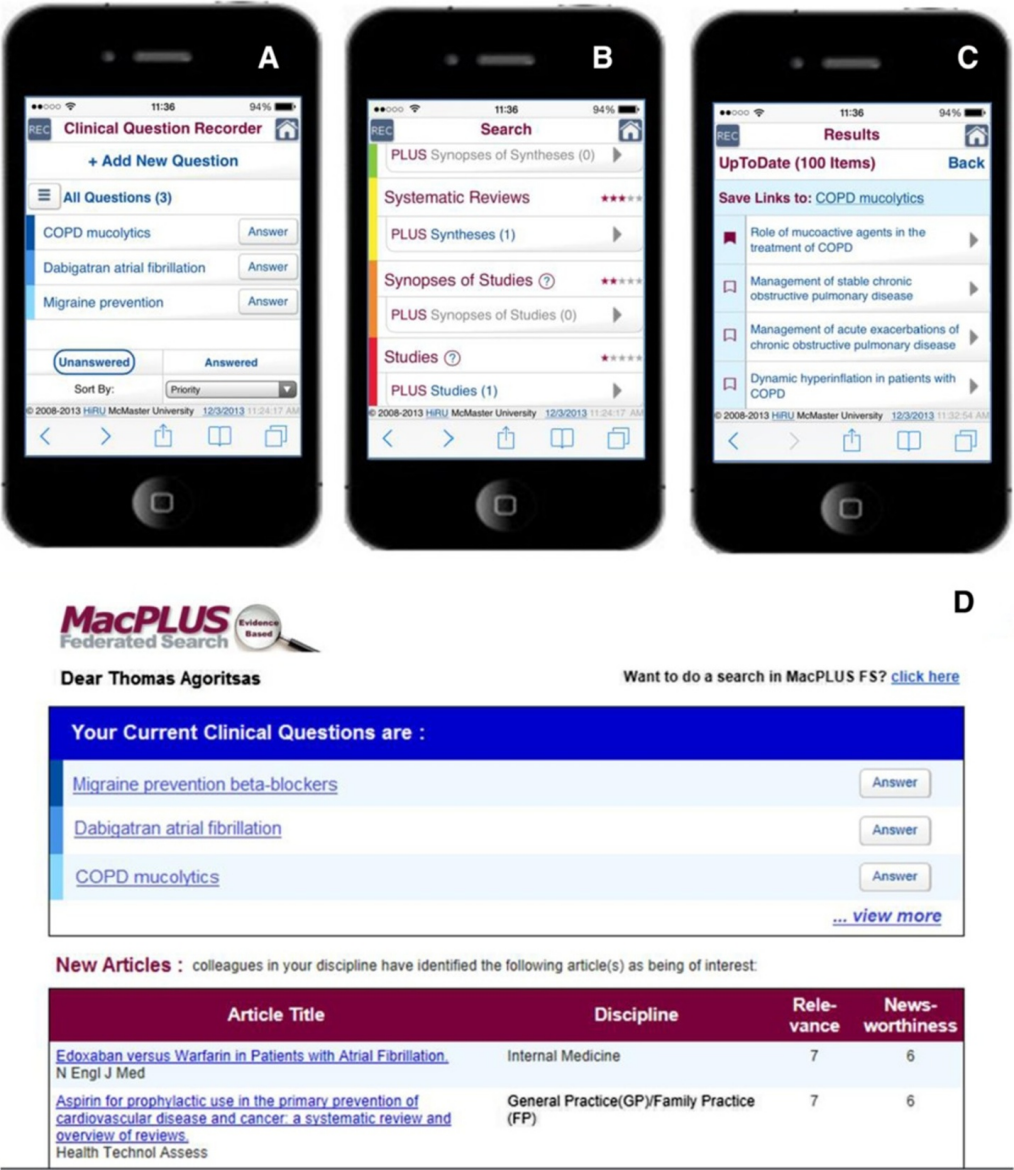
**Illustration of the Clinical Question Recorder and Reminder. A**,**B**,**C**,**D**: For a detailed description of each feature displayed, see the result section in the section "Intervention A - clinical question recorder".

The main features of the final Clinical Question Recorder are listed in Table 4 and illustrated in Figure 2. By simply clicking on `Add New Question,’ clinicians can type in and record their clinical questions directly on the web-based interface (Figure 2A). Clicking the `Answer’ button next to each question triggers a comprehensive search in MacPLUS FS according to the pyramid of EBM resources (Figure 2B). Links to relevant evidence can be bookmarked and saved with each clinical question for subsequent access and reading (Figure 2C), along with clinicians’ short answers. Periodic reminders of the list of unanswered questions are sent on top of regular MacPLUS FS alerts to new evidence (Figure 2D) - clicking on them or the `Answer’ button similarly triggers a search in MacPLUS FS.

**Table 4:** **Description of the features available in the three interventions**

**A**	**Clinical Question Recorder** (See also Figure 2)	Web-based interface, linked to MacPLUS FS account, and accessible on any smartphone, tablet and desktop computer.
		Easy recording and listing of clinical questions.
		Clicking the `Answer’ button next to each question triggers a comprehensive search in MacPLUS FS.
		Browsing of citations retrieved according to the pyramid of EBM resources.
		Bookmarking of links to relevant citations, saved along with the question.
		Recording of short answer to the question.
		Organizing of questions: setting priorities, sorting and classifying into folders.
		Reminders and links to unanswered questions are sent on top of regular MacPLUS FS alerts to new evidence.
		Answered questions and bookmarked evidence are saved and accessible in a virtual logbook of EBM practice.
**B**	**Evidence Retrieval Coach** (See also Figure 3)	Composed of eight short videos, embedded in MacPLUS FS.
		Display is tailored to clinician’s patterns of behaviors according to predefined triggers, or sent on a weekly basis as the trial unfolds.
		The title of each video (and gist of their content) are the following:
		1. MacPLUS FS - Why use it? (Answering questions with information overload)
		2. Enhancing Evidence-Based Clinical Practice (Using a parallel search in pre-appraised resources)
		3. A pyramid of resources (Overview of the architecture of evidence)
		4. Is one summary enough? (Top layers: Summaries)
		5. New and critically appraised evidence (Middle layers: Pre-appraised research)
		6. PubMed & the Clinical Queries (Bottom layers: Non-pre-appraised research)
		7. Preparing searchable questions (Using the PICO framework)
		8. Academic work (Using a federated search for presentations, grants and research)
**C**	**Audit, Feedback & Gamification** (See also Figure 4)	Allocation of badges, popping up online after a specific desired behavior, and also sent by email (about 50 badges available).
		Each badge is associated with an increase in reputation score, depending on the desirability of the behavior.
		It also provides a short, positively-framed feedback on the behavior, the number of times it was allocated to peers, and an upgraded reputation score.
		Clicking on the badges lead to a Reputation tab in MacPLUS FS providing the following features:
		Comparison of reputation with peers using pictographs (percentiles);
		List of badges obtained, clicking on them displays the full badge again;
		Graphical representation of daily reputation;
		Frequency of access to each EBM resources and mapping according to the pyramid.

### Intervention B - evidence retrieval coach

#### Development methods, feedback and usability

The purpose of this intervention is to facilitate the retrieval of current best evidence by providing guidance, `small bites’ of knowledge and skills through short videos. These videos are both embedded in MacPLUS FS and sent via e-mails according to each the clinician’s specific patterns of utilization and search.

We started this development by identifying specific teaching content that may help clinicians to benefit from available EBM resources in finding current best evidence. For that, we built on the strong expertise of our multi-disciplinary team in the Health Information Research Unit (HiRU), which has been one of the leading groups in evidence processing and retrieval, has contributed to many top EBM information resources over the past two decades, and has conceived MacPLUS FS. We wrote short scripts and mock-ups, and worked closely with an instructional designer (MP) to optimize language and presentation and produce the short videos.

We then asked our eight user-testers to provide independent feedback, particularly on understandability, usefulness, and satisfaction with the content and presentation. After two iterations, the videos were implemented in MacPLUS FS. We then asked our testers to check online usability while using the platform remotely.

### Results: description of the final features

**Figure 3:**
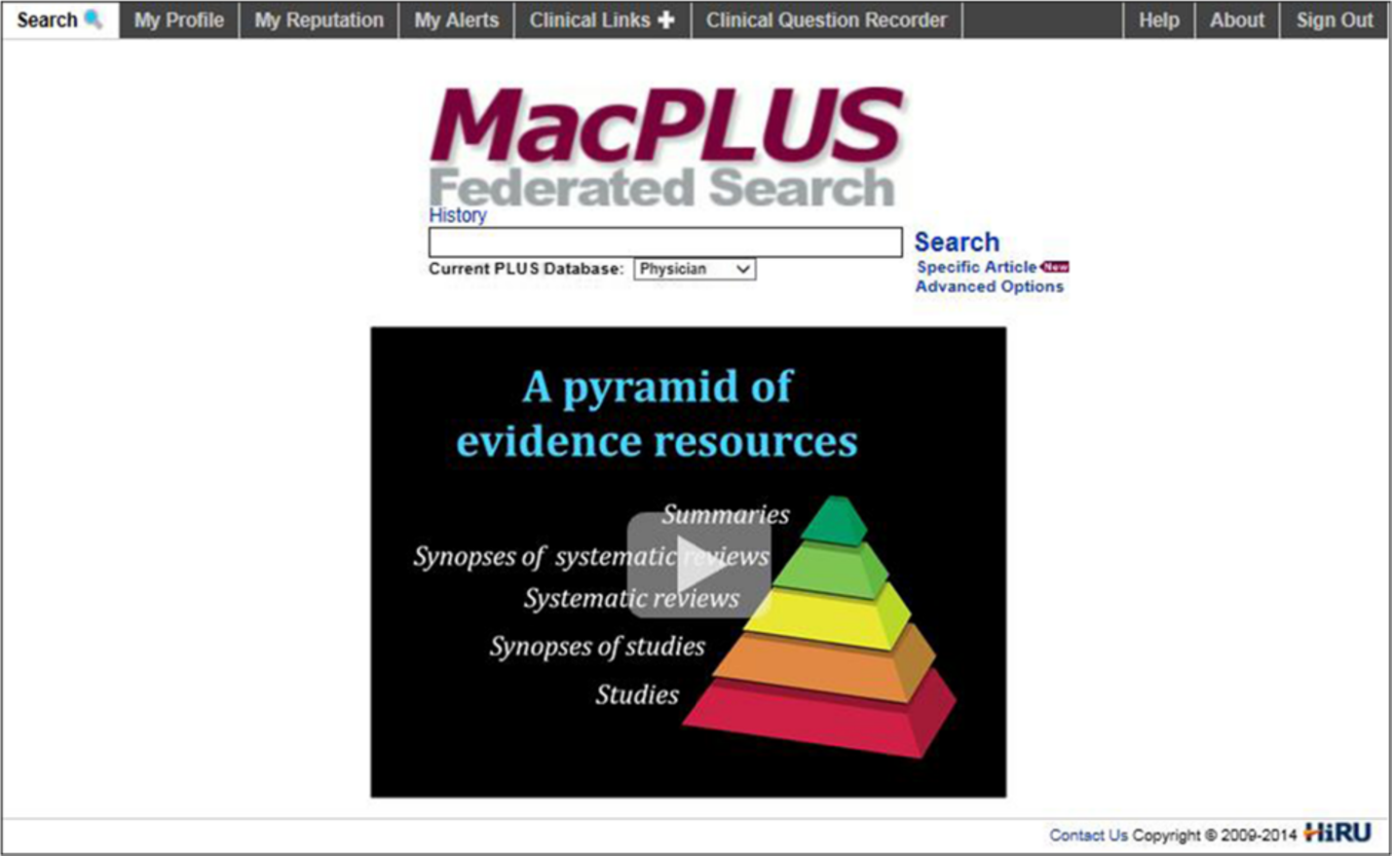
**Illustration of a video embedded in MacPLUS FS in the Evidence Retrieval Coach.**

The main features and the content of the videos within the Evidence Retrieval Coach are listed in Table 4. The intervention is composed of eight short videos lasting less than one and a half minute each. The videos are embedded in MacPLUS FS and accessible on smartphones, tablets and desktop versions (see Figure 3). The content covered includes an overview of the `architecture’ of evidence (pyramid), advantages and limits of individual resources (see Table 1), and how MacPLUS FS’s unique features overcome these limits and save time and effort (parallel comprehensive search, critical appraisal, organized presentation of complementary evidence). Special emphasis is put on showing how MacPLUS FS can be used for real-life evidence-based practice (*e.g*., to translate clinical questions and rapidly get reliable answers).

Moreover, the display of the videos is tailored to clinician’s individual patterns of behaviors, according to predefined triggers (see Additional file 2). After clinicians watch a video, they will receive its link by e-mail as an opportunity to watch it again later. These e-mails will be sent also on a weekly basis as the trial unfolds.

### Intervention C - audit, feedback and gamification

#### Development methods, feedback and usability

Based on behavioral theory, the purpose of this third intervention is to provide clinicians with timely feedback on their current search utilization compared to their peers. However, in a recent Cochrane review on 140 randomized trials, this approach showed only a 4.3% absolute increase in compliance with desired practice (95% CI 0.5% to 16%), with feedback being more effective when baseline performance is low and when it is provided regularly [39]. In light of these results, we decided to combine an audit and feedback intervention with a gamification approach [40], based on allocation of badges popping-up immediately after a desired behavior. These badges result in reputation scores that can be compared to peers on an interactive and playful interface within MacPLUS FS. Such approaches can enhance utilization and learning based on people’s natural desires for `competition, achievement, self-expression, and closure,’ and has been successfully used in many other educational settings [40].

We designed the online interface, badges and graphical presentation with the help of a user experience designer (AC). After internal usability testing of the features implemented, we asked our eight user-testers to evaluate the intervention while using the platform remotely, and provide independent feedback on usability, understandability, and satisfaction with the content and presentation.

### Results: description of the final features

**Figure 4:**
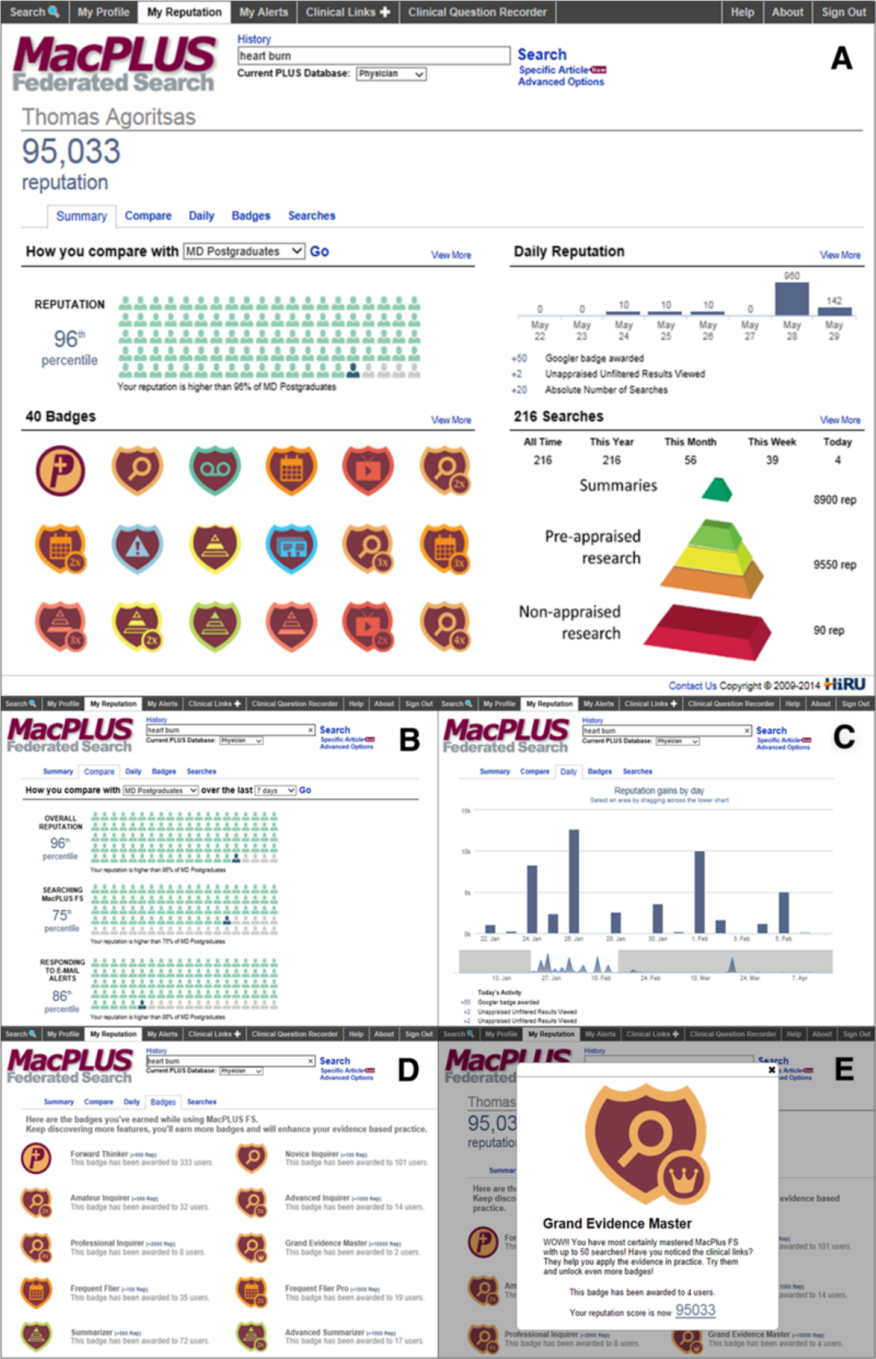
**Illustration of the components of the Audit, Feedback & Gamification. A** - **E**: For a detailed description of each feature displayed, see the result section in the section "Intervention C - audit, feedback and gamification.

The main features of the final audit, feedback and gamification interventions are listed in Table 4 and illustrated in Figure 4. All features are accessible within MacPLUS FS on a `reputation tab’ (Figure 4A). We generated about 50 badges rewarding the following behaviors: total and weekly frequencies of searches, frequencies of access to the top layers of the EBM resource pyramid (summaries), to the middle layers (pre-appraised research), and to bottom layers (non-pre-appraised research), number of complementary resources accessed per search, number of alerts to new evidence accessed, number of questions recorded (for users also allocated to the Clinical Question Recorder), and number of videos watched (for those allocated to the Evidence Retrieval Coach).

Each badge was assigned a reputation score based on the desirability of the behavior it reinforces. Badges pop-up online after a specific behavior (Figure 4E), award their reputation score to the user, and can be accessed again later (Figure 4D). Clinician’s resulting reputation score can be compared to peers’ through percentiles displayed in interactive pictographs (Figure 4B), and followed graphically across time (Figure 4C). Finally, clinicians can explore their access to each EBM resource, mapped according to the EBM pyramid (Figure 4A).

### III. Protocol of the randomized controlled trials

#### Setting and study participants

We will conduct the trials described in this protocol in the teaching hospitals and clinics affiliated with McMaster University, Ontario, Canada. This amounts to 2 major academic hospital systems, operating 10 hospitals in the Hamilton area, as well as 2 regional campuses in Niagara and Waterloo, Ontario.

Currently about 2,800 clinicians and students are registered in MacPLUS FS. The first trial will be conducted among all postgraduate trainees, and the second trial among all faculty registered in MacPLUS FS at the beginning of the trials, after exclusion of those no longer physically working at McMaster University affiliated hospitals. We will also exclude registrants who have never interacted with MacPLUS FS, either by logging in to read email alerts or to perform a search, during the last 12 months counting back from the beginning of the trials, regardless of how long they have been registered. These broad eligibility criteria reflect our choice to perform pragmatic effectiveness trials, rather than focusing only on high-frequency users. Indeed, our objective is precisely to increase the quantity and quality of searches among low-frequency users in real clinical practice. Nevertheless, we are excluding registrants with a very high probability of being unexposed or insensitive to our web-based interventions, either because they are no longer at our institution or have repeatedly ignored MacPLUS FS over a prolonged period.

By December 31, 2013, these eligibility criteria were met by 904 clinicians - 429 postgraduate and 475 faculty MDs (see Table 5) - after exclusion of 211 registrants no longer working at McMaster University, and 284 who never interacted with MacPLUS FS during the last year. About two-thirds of eligible users interacted with MacPLUS FS only through email alerts, while one-third performed at least one search in that period. About 16% of eligible clinicians work in the field of internal medicine, 32% work in family medicine, while the other half of the sample works in a wide array of other specialties (see Table 5).

**Table 5:** **Baseline utilization among the eligible 904 MDs during the six months prior to the trial**

	Postgraduates	Faculty	Total MD
	(n = 429*)	(n = 475*)	(n = 904*)
**Specialty type** - n (%)			
Internal Medicine	82 (19.1%)	66 (13.9%)	148 (16.4%)
Family Medicine	107 (24.9%)	184 (38.7%)	291 (32.2%)
Other Specialties	240 (55.9%)	225 (47.4%)	465 (51.4%)
**Total number of searches**	935	423	1,358
**Searches/month/user** - Mean (SD)	0.46 (1.42)	0.20 (0.83)	0.32 (1.16)
**Categories of search frequency** - n (%)			
>5 *(Super-searchers)*	8 (1.9%)	4 (0.8%)	12 (1.3%)
1 to 5 *(Regular-searchers)*	45 (10.5%)	24 (5.1%)	69 (7.6%)
<1 *(Occasional-searchers)*	89 (20.7%)	72 (15.2%)	161 (17.8%)
0 *(Alert-only-users)*	287 (66.9%)	375 (78.9%)	662 (73.2%)
**Time since last search** - n (%)			
<= 365 days	163 (38.0%)	143 (30.1%)	306 (33.8%)
>365 days	266 (62.0%)	332 (69.9%)	598 (66.2%)
**Total number of e-mail alerts read**	4,064	7,092	11,156
**E-mail alerts read/month/user** - Mean (SD)	1.65 (2.99)	2.54 (6.03)	2.12 (4.85)
**Total number of other weblogins**	1163	740	1903
**Other weblogins/month/user** - Mean (SD)	0.52 (4.10)	0.32 (2.85)	0.41 (3.50)

*Four additional participants (two postgraduates and two faculty) are missing from this count, as they registered in Jan 2014, just before the beginning of the trial.

### Randomization

Participants will be randomized to our three web-based interventions in a factorial design (see overview of study design and Table 2). Postgraduates and faculty MDs will be randomized separately and further stratified according to time since last search (<= 365 days vs. >365 days; see Table 5), as an overall proxy of their baseline frequency searches in MacPLUS FS. Right before the beginning of the trials, participants will be randomly allocated to each factorial group (2^3^ = 8 groups), balancing on blocks of 16 within each stratum (=2 - 8). Our information technology programmers, in charge of MacPLUS FS system administration, will perform randomization using a computer-based pseudo-random number generator. They will maintain a secure master list of the randomization codes and assignments, and conceal allocation from the analysts.

### Blinding and control group

Although participants cannot be blinded to the interventions, they will not be informed of the different interventions that are being offered. In addition, all participants, including the control group with no intervention, will be exposed to new minor features one month prior to the beginning of the trial. These include: small changes in the web design (simplification of available tabs and navigation), waiting time features displaying all resources searched in parallel in MacPLUS FS (see Figure 1), and a novel `single citation matcher’ (see Table 1). These minor new features would thus further minimize the risk of contamination between the intervention arms from users becoming aware of interventions they are missing. Moreover, the interventions cannot be shared, as they are linked to individuals’ accounts, so that it is unlikely that registrants who are not offered an intervention would increase their utilization just by hearing about it.

### Outcomes

#### Primary outcome

Our primary question is whether each intervention increases the quantity of searches to answer questions - *i.e*., search utilization (not counting logins to e-mail alerts or to access other resources). This will be measured by (i) rate of searches/month/user, (ii) and corresponding proportions of `super-searchers’ (>five searches/month), `regular-searchers’ (one to five searches/month), `occasional-searchers’ (<one search/month), and `alert-only-users’ (no searches/month). The primary outcome will be averaged over six months, but continuously recorded as participants will be signed on through their individual user account that tracks logins and use of EBM resources, down to individual keystrokes.

Table 5 shows the baseline utilization data during the six months prior to the start of the trial, from July to December 2013. Postgraduates MDs (n = 429) searched MacPLUS FS 935 times in total, corresponding to about 0.46 searches/month/user, whereas they accessed 4,064 alerts to new evidence, corresponding to 1.65 alerts/month/user, and consulted other web-resources in MacPLUS FS 0.52 times/month/user. About 66.9% of postgrads users were `alert-only-users,’ while 10.5% were `regular-searchers’ and 1.9% `super-searchers.’

The utilization patterns were different for Faculty MDs (n = 475) who searched MacPLUS FS half as much, about 423 times in total, corresponding to about 0.20 searches/month/user, whereas they accessed almost twice as many alerts to new evidence, 7,092 alerts in total, corresponding to 2.54 alerts/month/user, and consulted other web-resources in MacPLUS FS 0.32 times/month/user. About 78.9% of faculty used were `alert-only-users,’ while 5.1% were `regular-searchers’ and 0.8% `super-searchers.’

### Secondary outcomes and questions

We will assess whether each intervention can increase the utility of the evidence retrieved (satisfaction in meeting users’ information needs, expected impact on one’s general practice), the use of the evidence (the extent of use when caring for a specific patient), and its perceived usefulness in patient care and outcomes (perceived benefits of applying the evidence for a specific patient). Utility, use and usefulness of the evidence retrieved will be assessed using an adapted version of the Impact Assessment Method (IAM) [41]-[43], which was specifically developed for assessing how clinicians use information, based on the Acquisition-Cognition-Application-Outcome Model [42]-[44]. This validated six-item questionnaire takes less than one minute to complete online and will be sent by e-mail for online completion following a pre-defined automatic algorithm. The first invitation will be sent out one month after the participant’s first online exposure to one or more interventions, with one reminder after 24 hours. The next invitation will be sent following the next search, but after a two-week delay. This process will be repeated until one filled questionnaire for a clinical question is returned, or the trial ends (see details and full questionnaire in Additional file 3). Perceived usefulness will be analyzed as the `number needed to benefit from evidence,’ defined as the number of patients for whom the evidence has to be retrieved to observe or expect health benefits for one patient [45]*.*

Other secondary questions that we plan to address include whether each intervention efficacy varies across time within the six-month trial (*e.g*., persistent, transient, increasing or decreasing effect), and whether the interventions have an impact on non-searching utilization of MacPLUS FS (*i.e*., frequency of alerts read, frequency of web logins for other clinical resources).

Finally, we will explore if the interventions modify the patterns of use of the different EBM resources, and in particular if they increase the accesses to higher levels of evidence, such as summaries and pre-appraised research, compared to non-pre-appraised research. Table 6 displays the baseline distribution of access across the pyramid of EBM resources among clinicians that have adopted MacPLUS FS, that is, `regular-searchers’ and `super-searchers.’ With 1,025 searches, these users have conducted about 75% of all searches in MacPLUS FS, and accessed one of its resources 1,390 times in total. All resources in the federated search were consulted: summaries were accessed 53.2% of the times, pre-appraised research in 16.1%, and non-pre-appraised research in 30.6% of the times. Postgraduates searched less summaries than faculty did (49.0% vs. 63.8%), and more non-pre-appraised resources (35.3% vs. 19.3%).

**Table 6:** Baseline frequency of access to EBM resources (% of all accesses), among `regular-searchers’ and `super-searchers’*

		Postgraduates	Faculty	Total
		(n = 53)	(n = 28)	(n = 81)
		739 searches	286 searches	1,025 searches
**Summaries**		**485 (49.0%)**	**255 (63.8%)**	**740 (53.2%)**
	DynaMed	174 (17.6%)	39 (9.8%)	213 (15.3%)
	UpToDate	120 (12.1%)	128 (32.0%)	248 (17.8%)
	Best Practice	147 (14.8%)	71 (17.8%)	218 (15.7%)
	ACP PIER	44 (4.4%)	17 (4.3%)	61 (4.4%)
**Pre-appraised research**		**156 (15.8%)**	**68 (17.0%)**	**224 (16.1%)**
	Synopses of systematic reviews	23 (2.3%)	17 (4.3%)	40 (2.9%)
	Systematic reviews	66 (6.7%)	21 (5.3%)	87 (6.3%)
	Synopses of studies	10 (1.0%)	4 (1.0%)	14 (1.0%)
	Studies	57 (5.8%)	26 (6.5%)	83 (6.0%)
**Non-pre-appraised research**		**349 (35.3%)**	**77 (19.3%)**	**426 (30.6%)**
	Filtered studies	257 (26.0%)	60 (15.0%)	317 (22.8%)
	Unfiltered studies	92 (9.3%)	17 (4.3%)	109 (7.8%)
**Total number of accesses**		**990 (100%)**	**400 (100%)**	**1,390 (100%)**

*i.e., clinicians who conducted more than one search per month on average.

### Hypotheses and statistical analysis

The two trials are separate and will be analyzed as such. We have three primary hypotheses for each trial: that the clinical question recorder will be more effective than the control; that the evidence retrieval coach will be more effective than the control; and that audit, feedback and gamification will be more effective than the control. Each of these hypotheses will be tested separately (half of the sample compared to the other half). The effect of each intervention will be tested by regressing the average number of searches per month over the trial’s six-month time period for each user onto dummy variables for each intervention, controlling for search frequency at baseline. The distribution of the number of searches per user is not known at present, but baseline data suggests excess zeros with extra-Poisson variation. We will attempt to capture the distribution parametrically, but in the event that it is not possible to do this accurately, we will use ordinary least squares to estimate the regression coefficients together with heteroscedasticity-robust standard errors.

### Potential subgroup effects

Prior to the start of the trial, we hypothesized that the impact of the intervention on our primary outcome may differ according to specialty type - *e.g*., more effective in clinicians practicing internal medicine than family medicine or other specialties - and according to baseline frequency of search during the six months prior to the trial - *e.g*., higher frequency searchers would tend to be more responsive to each intervention (see Table 5 for the baseline data for these two pre-specified subgroups). In an exploratory analysis, we will test for subgroup effects, using tests of interactions between the dummy variables for intervention and subgroup variables.

### Potential interactions between the interventions

Our primary analysis will be at the margins, that is, looking at each effect independently, but we will also test for interactions among the interventions. We expect that combining them will have an additive effect, and that an interaction is unlikely, particularly a sub-additive one (*e.g*., one intervention being effective alone, but less effective or even ineffective in combination with another). We cannot formally exclude any synergistic interaction (beyond additivity), but we have no reason to expect it *a priori*[46]. Moreover, observing a synergistic effect would not jeopardize our results, as we are more interested in finding any `signal’ of effect of the interventions, rather than estimating their independent effect with maximal accuracy. By analogy with drug trials, this study would be a phase II rather than a phase III randomized trial, given the current state of research in the field.

### Power calculation

Since we anticipated that interactions among the interventions are unlikely, we have powered the trials assuming no such interactions. Before the trials began, we had 904 participants eligible for the study, of whom 429 were postgraduates and 475 were faculty (see Table 5). Baseline data indicated a mean of 0.46 searches per month per user (SD 1.42) among postgraduates and 0.20 searches per month per used (SD 0.83) among faculty. Additional file 4 shows power curves for the faculty and for the postgraduates. These indicate that among the postgraduates, we will have 80% power to detect an increase of 0.9 in the mean number of searches per month, and among the faculty we will have 80% power to detect an increase of 0.5 in the mean number of searches per month.

### Analysis of secondary questions

An exploratory analysis will investigate time trends in intervention efficacy. Rather than using the average number of searches per user per month over the six months of the trial, we will conduct a longitudinal analysis using the number of searches per user for each of the six months of the trial as the dependent variable, regressed onto time, dummy variables for each of the interventions, the interaction between time and intervention, together with search frequency at baseline. This regression model will be fitted using a Generalized Estimating Equation (GEE).

Finally, we will compare the distribution of answers on the IAM questionnaire *(i.e*., utility, use and usefulness of the evidence retrieved), as well as the distribution of access to the different EBM resources, using chi-squared tests.

### Ethics and registration

Upon registration to MacPLUS FS, users will consent to participate in its evaluation. Namely, they will agree that their use of MacPLUS FS will be measured for frequency and type of use, and that they will receive periodic online evaluation questionnaires. No individual identifiers will be stored in the monitored databases. The Hamilton Integrated Research Ethics Board has approved this project (REB Project #05-186), as well as a specific waiver for additional informed consent for registrants to be randomized to the different interventions, as no risk is involved and it is necessary to preserve blinding to provide an unbiased utilization measurement (primary outcome). The trials have been registered at ClinicalTrials.gov before randomization (ClinicalTrials.Gov NCT02038439).

### Trial administration and data management

The trials will be conducted at the Health Information Research Unit, at McMaster University, which designed and is hosting MacPLUS FS. Before the trials start, research staff (EI) and the principal investigator (TA) will check eligibility criteria of the registrants, verify their affiliation to McMaster, profession and training level, and crosscheck the information stated at registration with official administrative medical databases.

The administration of interventions, outcome measurements, and the sending of periodic online IAM questionnaires will all be programmed before randomization and further handled automatically as they will be built into the MacPLUS FS online infrastructure.

The trials will start simultaneously for all participants. All interactions with MacPLUS FS, including any click-through links within emails, will automatically sign participants on through their individual user account that tracks logins and use of EBM resources. Primary and secondary outcomes will be recorded from this automatic monitoring of the system, and stored in a specific and secure database within MacPLUS FS.

The research staff (EI) and the principal investigator (TA) will review overall utilization data collected on a weekly basis, looking for completeness of data and navigational bugs. However, no interim analysis will be performed before the trial end.

## Trial status

The trial is currently ongoing at the time of submission of this manuscript. We have not begun and will not perform any data cleaning, analysis or interim reports before the trial ends.

## Discussion

The three interventions in these two factorial randomized trials are innovative in at least three different ways. First, although widely used in other fields such as education, task management, business, or customer user-centered services, we are not aware that any of these approaches have been applied thus far to clinical evidence retrieval. Second, the interventions use web-based technology to facilitate low cost implementation at a broad scale, for all types of devices. Smartphones and tablets are transforming the way we live, practice medicine, and intuitively learn new skills [47],[48]. Third, the general framework for these interventions is based on effective models for teaching clinical skills at the point of care. These models have changed the way we teach clinical examination or diagnostic reasoning - embedded in our daily practice [30]-[34] - but have not yet been used to teach how to find current best evidence in the point of care, a skill that has nevertheless become designated `as essential as the stethoscope’ [8].

Our trials have also inherent limitations. First, although MacPLUS FS includes most top EBM resources currently widely used by clinicians (see Table 1), participants may still opt to access individual resources directly rather than through MacPLUS FS. However, although this may result in an apparent low frequency of search, the randomization should balance the distributions of such behaviors across study arms and not jeopardize the conclusion from the trials.

Second, although the validated IAM will try to capture the secondary outcomes of utility, use and perceived usefulness of the retrieved, these surveys may suffer from suboptimal response rates. Ideally, we would assess the effect on directly measured patient important outcomes, but this is beyond the feasibility of the current study, and particularly challenging at hospital-levels across a very wide array of potential clinical questions. In any event, the justification for doing a larger multi-centered trial with direct measurement of patient outcomes would be the observation of a sufficient utilization rate associated with a substantial effect on evidence use and usefulness in the present trial. By analogy with drug trials, this study would be a phase II trial.

Third, the interventions are primarily mediated through emails with direct login access to MacPLUS FS, and as such, their potential impact may be diluted in the numerous competing solicitations clinicians continuously receive through emails. Moreover, the Clinical Question Recorder (Intervention A) requires clinicians to actively record their questions. To maximize the chances they engage, we focused our efforts upstream in the user-centered design, implementation and testing of the recorder. Actual use in real life settings remains uncertain, although simply offering the intervention may also have some indirect effect on searches.

Finally, baseline data showed that search rates heavily fluctuate across time. Lower rates at certain periods (*e.g*., holidays, vacation days, or exam periods) may affect the assessment of the interventions, although utilization averaged over six months of follow-up should allow a reasonable comparison between study arms.

The main advantages of this study rely on the feasibility of the administration of the interventions and the outcomes measurements for a large number of clinicians, as these will be handled automatically in MacPLUS FS online system, with no possibility of crossover, and virtually no loss of follow-up for primary outcome data.

In conclusion, the trials will answer whether these innovations have the potential of enhancing knowledge translation through a clinician’s timely access to current best evidence. The MacPLUS FS interface allows a broad implementation for registrants, in a sustainable way, with limited additional costs. If effective, these interventions can further be broadly implemented beyond the McMaster community, using the twin version of MacPLUS FS - called ACCESSSS FS (http://plus.mcmaster.ca/ACCESSSS), and enhance the access to current best evidence for a large audience, across many top EBM resources in parallel, and tied directly to clinical questions.

## Authors’ contributions

Conception of the interventions and study design: TA, with significant contributions from BH, NW, and AI. Specific design of intervention A (Clinical Question Recorder): TA and NH. Specific design of intervention B (Evidence Retrieval Coach): TA, MP, EI and PR. Specific design of intervention C (Audit, Feedback & Gamification): TA, NC, AC. IT programming: NH, RP and CC. Conduct of the user-testing: TA. Analysis of baseline data: TA and EI. Statistical analysis plan: TA and EP. Overall supervision: BH. Draft of the manuscript: TA. Critical revision of the manuscript for important intellectual content: EI, NH, NC, AC, PR, MP, CC, RP, EP, NW, AI and BH. All authors read and approved the final manuscript. TA had full access to all the data presented in the manuscript and takes responsibility for its integrity and accuracy.

## Additional files

## Electronic supplementary material

Additional file 1: User-testing interview guide for the development of the Clinical Question Recorder.(PDF 262 KB)

Additional file 2: Evidence Retrieval Coach: tailoring the educational videos to clinicians pattern of use.(PDF 77 KB)

Additional file 3: Online administration of Impact Assessment Method (IAM) questionnaire.(PDF 191 KB)

Additional file 4: Power curves for the primary outcome.(PDF 48 KB)

Below are the links to the authors’ original submitted files for images.

Authors’ original file for figure 1

Authors’ original file for figure 2

Authors’ original file for figure 3

Authors’ original file for figure 4

## Abbreviations

EBM: Evidence-based medicine
MacPLUS FS: MacPLUS federated search
PLUS: Premium literature service
IAM: Impact assessment method
PICO: Population - Intervention(s) - Comparator - Outcome(s)
