# Supplementary material for: Increasing the quantity and quality of searching for current best evidence to answer clinical questions: protocol and intervention design of the MacPLUS FS Factorial Randomized Controlled Trials
Source: Implement Sci. 2014 Sep 20;9:125. doi: 10.1186/s13012-014-0125-9 (PMC4177052; doi:10.1186/s13012-014-0125-9)
Supplement: Supplementary file 1 — Additional file 1: User-testing interview guide for the development of the Clinical Question Recorder.(PDF 262 KB) [file 13012_2014_125_MOESM1_ESM.pdf]

# Additional file 1. User-testing interview guide

## MacPLUS FS Trial - Clinical Question Recorder (Intervention A)

*Adapted from templates available at http://www.usability.gov, U.S. Dept. of Health and Human Services. The Research-Based Web Design & Usability Guidelines, Enlarged/Expanded edition. Washington: U.S. Government Printing Office, 2006.*

**Notetaker**: **Location / Date:** **Iteration**:

**Participant #** **Initials**: **Gender** :

**Profession/Background**:

**Type of smarphone**:

## Test Facilitator Guide:

### Welcome and Purpose

Thank you for agreeing to participate in the evaluation of this App. Today I am asking you to serve as an evaluator of this App and to complete a set of scenarios. My goal is to see how easy or difficult you find the App to use. We will record your reactions and opinions; so, we may ask you to clarify statements that you make from time to time.

### Test Facilitator's Role

I'm here to record your reactions and comments of the App you'll view. During this session I will not be able to offer any suggestions or hints. There may be times, however, when I'll ask you to explain why you said or did something.

### Test Participant's Role

I will ask you to search for information on this App to learn if it works well for you. We'll do this by giving you scenarios or tasks to complete on the site. You also will be asked a series of questions about your experience at the end of this session.

### Things to Keep in Mind

Here are some things that you should know about your participation:

- This is not a test of you; you're testing the App. So don't worry about making mistakes.
- There is no right or wrong answer. We really just want to know if we designed the App well for you.
- If you ever feel that you are lost or cannot complete a scenario with the information that you have been given, please let me know. I'll ask you what you might do in a real-world setting and then either put you on the right track or move you on to the next scenario.
- (We will be audio recording this session for further study if needed. Your name will not be associated or reported with data or findings from this evaluation. Please fill out the audio release form.)
- Finally, as you use the App, please do so as you would in real life. I do ask that when looking for information, you do so as quickly and as accurately as you can.

Do you have any questions before we begin?

## Usability features to be tested:

1. Connect  through your smartphone
2. Explore the CQR App.
3. Enter a clinical question
4. Answer a clinical question
5. Set/change priority for a question
6. Select relevant citations
7. Record an answer
8. Create a folder
9. Find again an old question and re-run a search

---

## A. Pre Test Questions

- Just from looking at this Clinical Recorder App, what do you expect to find in it? What usefulness to you expect? Please be specific.

- Who do you think this site is designed for?  Why? (Probe: MD, nurses, undergrad, postgrad, faculty, rehab)

- Have you ever used some similar App? Which one and in what context?

- If you were to envision your ideal CQR App, what features should it have? What would it look and act like? How would it be organized?

## B. Participant Tasks

### Task 1

- Can you access the Clinical Question Recorder with the link you've been provided?
- Can you create an icon on your desktop to access it more easily later.

| **Pathway(s)** | **Success (Circle 1)** | **Notes/Observations** (Note why was the user successful or not successful, e.g., wrong pathways, confusing page layout, navigation issues, terminology) |
|---|---|---|
| Connect through your smartphone<br><br>Create an icon on the phone?<br><br><u>Optional</u><br><br>Find MPFS (google)? | 0<br>Not completed<br><br>1<br>Completed with difficulty or help<br><br>2<br>Easily completed | |

### Task 2

- Can you explore the App intuitively, without any instruction. Please describe what you see and "think aloud" ("What's this button? I wonder? When I click here…"). Be specific.

| **Pathway(s)** | **Success (Circle 1)** | **Notes/Observations** (Note why was the user successful or not successful, e.g., wrong pathways, confusing page layout, navigation issues, terminology) |
|---|---|---|
| Explore the app, can go as far as create a question and answer. | 0<br>Not completed<br><br>1<br>Completed with difficulty or help<br><br>2<br>Easily completed | |

## Task 3

- You are an MD student doing your internship in internal medicine. During the rounds, you examine a patient with a facial paresis. Your instructor tells you it is a Bell palsy and you discuss with her its specific signs and diagnostic procedure. You then wonder what are the treatment options, but have no time to do an extensive search right now.
- Using the Clinical Question Recorder App, can you record your question. When you feel you have completed this task, please say so.

| **Pathway(s)** | **Success (Circle 1)** | **Notes/Observations** (Note why was the user successful or not successful, e.g., wrong pathways, confusing page layout, navigation issues, terminology) |
|---|---|---|
| Add new question<br>Enter question (edit question)<br>Type search terms/full question?<br>Save<br>Navigate back<br><br><u>Optional</u><br><br>Set priority?<br>Choose folder (if already exists)? | 0<br>Not completed<br><br>1<br>Completed with difficulty or help<br><br>2<br>Easily completed | |

## Task 4

- Later after Lunch, you remember  your question about the treatment of Bell palsy. You decide do go back in the recorder and answer your question.

| **Pathway(s)** | **Success (Circle 1)** | **Notes/Observations** (Note why was the user successful or not successful, e.g., wrong pathways, confusing page layout, navigation issues, terminology) |
|---|---|---|
| Click Answer<br>Goes to MPFS Phone version<br><br>Assess (in)adequacy of search terms<br><br><u>Optional</u><br><br>Explore results from the pyramid<br>Bookmark options. | 0<br>Not completed<br><br>1<br>Completed with difficulty or help<br><br>2<br>Easily completed | |

## Task 5

- In the afternoon the same day, you follow a fellow during outpatients consultations. Several questions occur and you use the Clinical Question Recorder to record them.
- Questions include the prevention of migraine, the usefulness of mucolytics for COPD patients, the use of rivaroxban for the prophylaxis of thrombo-embolic events in cancer patients.
- You wonder if you can quickly classify them by priority? And sort them alphabetically?

| **Pathway(s)** | **Success (Circle 1)** | **Notes/Observations** (Note why was the user successful or not successful, e.g., wrong pathways, confusing page layout, navigation issues, terminology) |
|---|---|---|
| Edit questions<br><br>Set priority<br><br><u>Optional</u><br><br>Explore sorting options? | 0<br>Not completed<br><br>1<br>Completed with difficulty or help<br><br>2<br>Easily completed | |

## Task 6

- To answer your question about migraine prevention, you rapidly screen citations retrieved by MacPLUS FS. Can you select the one that are potentially relevant? Can you do that for other questions?

| **Pathway(s)** | **Success (Circle 1)** | **Notes/Observations** (Note why was the user successful or not successful, e.g., wrong pathways, confusing page layout, navigation issues, terminology) |
|---|---|---|
| Click + sign to bookmark<br><br>Bookmark on different pyramid layers.<br><br>Navigate back to the question display to see saved citations.<br><br><u>Optional</u><br><br>Sorting options? | 0<br>Not completed<br><br>1<br>Completed with difficulty or help<br><br>2<br>Easily completed | |

## Task 7

- After a quick reading of 1 min, you want to answer your question in a short note. Do so in the Clinical Question Recorder. After you're done, can you edit your question?

| **Pathway(s)** | **Success (Circle 1)** | **Notes/Observations** (Note why was the user successful or not successful, e.g., wrong pathways, confusing page layout, navigation issues, terminology) |
|---|---|---|
| Select question<br>Click unanswered<br>Type answer<br>Save<br><br>Optional<br><br>Edit the question? | 0<br>Not completed<br><br>1<br>Completed with difficulty or help<br><br>2<br>Easily completed | |

## Task 8

- At the end of the day, your supervisor tells you would have to present a topic at the end of the month. You think 2-3 of the questions you've recorder might be a good topic. Can you create a folder named "presentation" and class your question within?

| **Pathway(s)** | **Success (Circle 1)** | **Notes/Observations** (Note why was the user successful or not successful, e.g., wrong pathways, confusing page layout, navigation issues, terminology) |
|---|---|---|
| Create a folder<br><br>Click on folder symbol<br>Add new folder<br>Enter a name<br>Save<br>Close<br><br>Navigate back and access the folder presentation<br><br>Optional<br><br>Class a question in the folder? | 0<br>Not completed<br><br>1<br>Completed with difficulty or help<br><br>2<br>Easily completed | |

### Task 9

- 3 weeks later, the time has come to make your presentation. Assuming there might be new evidence, you want to re-run the search for your question of interest

| Pathway(s) | Success (Circle 1) | **Notes/Observations** (Note why was the user successful or not successful, e.g., wrong pathways, confusing page layout, navigation issues, terminology) |
| --- | --- | --- |
| Find again an old question and re-run a search<br><br>Click unsanswered<br>Select a question<br>Click on search | 0<br>Not completed<br><br>1<br>Completed with difficulty or help<br><br>2<br>Easily completed | |

## C. Post Test Interview

- What is your overall impression to this CQR App?

- If you had to give the site a grade, from 10 to 0, where 10 was exemplary and 0 was failing, what grade would you give it, and why?

- What did you like best about the site?

- What did you like least about the site?

- Is there anything that you feel is missing on this site? (Probe: content or site features/functions)

- If you were the website developer, what would be the first thing you would do to improve the App?

- Would you recommend this App site to a colleague? To a friend?

- If you were to describe this App to a colleague in a sentence or two, what would you say?

- Would you use this App your own in the future? Why/why not?

- Do you have any other questions or comments about the Web site or your experiences with it?

# IDENTIFICATION OF ISSUES:

For each of the above scenarios, we will identify identifying major and minor problems and suggestions for improvements, according to the following coding:

**Xxx Show-stoppers**
**Xx Big problems/frustration (but eventually figured it out)**
**X Minor frustrations or cosmetic things**
**0 Positive feedback**
**00 Suggestions for improvement**
**ID Personal idea arising from testing**

We will further categorize these issues of users' experience according to **Peter Morville's revised honeycomb framework of user experience:**

> *Rosenbaum SE, Glenton C, Nylund HK, Oxman AD: User testing and stakeholder feedback contributed to the development of understandable and useful Summary of Findings tables for Cochrane reviews. J Clin Epidemiol 2010, 63:607-619.*

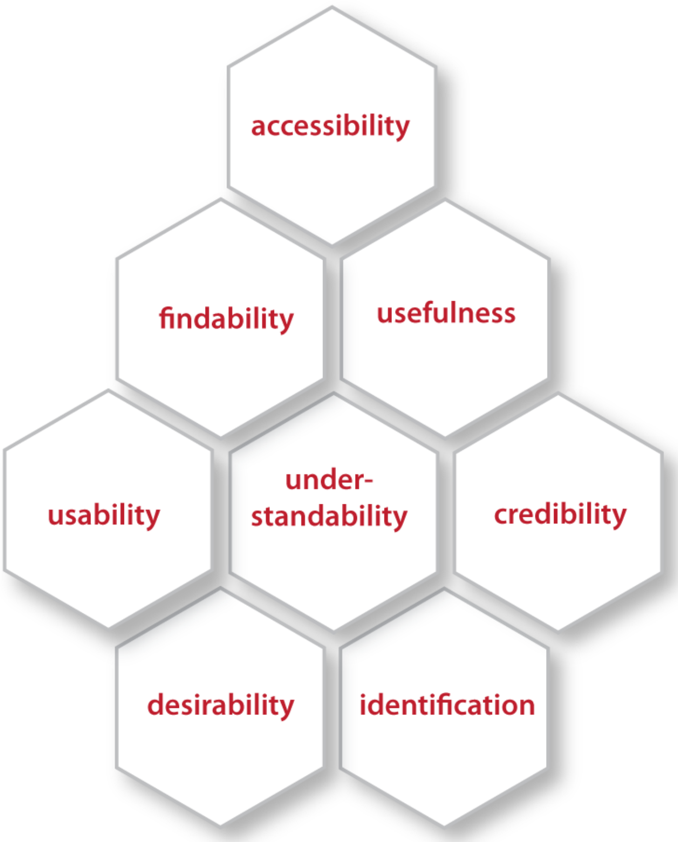


**Findability:** can this person locate the product or the content that they are looking for?

**Accessibility:** are there physical barriers to actually gaining access, also for people with handicaps, like color blindness?

**Usability:** how easy and satisfying is this product to use?

**Usefulness:** does this product have practical value for this person?

**Credibility:** is the product/content experienced as trustworthy?

**Desirability:** is the product something this person wants? Has a positive emotional response to?

**Understandability:** does this person comprehend correctly both *what kind* of product this is, and comprehend the content correctly? Is this person's subjective experience of whether or not they understand in line with their actual (correct or incorrect) understanding?

**Identification:** does this person identify with the product, on a personal or a social level? Or is it alienating, experienced as being not designed for "someone like me".
