# Supplementary material for: Increasing the quantity and quality of searching for current best evidence to answer clinical questions: protocol and intervention design of the MacPLUS FS Factorial Randomized Controlled Trials
Source: Implement Sci. 2014 Sep 20;9:125. doi: 10.1186/s13012-014-0125-9 (PMC4177052; doi:10.1186/s13012-014-0125-9)
Supplement: Supplementary file 2 — Additional file 2: Evidence Retrieval Coach: tailoring the educational videos to clinicians pattern of use.(PDF 77 KB) [file 13012_2014_125_MOESM2_ESM.pdf]

<u>Additional File 2</u>. Evidence Retrieval Coach: tailoring the educational videos to clinicians pattern of use

---

- The coach is composed of 8 short educational videos, lasting 1 to 1.30 minutes.
- Two types of triggers are implemented:
  - Specific triggers that try and tailor the display of the video to users specific behaviours
  - Weekly time triggers, as the trial unfolds.

Name of short educational video	1. Specific Triggers	2. Triggers
1. Why use it?<br>*[Answering questions with information overload]*	*No trigger, sent at by e-mail and visible on the search page at the beginning of the trial	Time 0
2. Enhancing Evidence-Based Clinical Practice<br>*[Using parallel search in pre-appraised resources]*	*Available on the "Clinical Vital Links"	After 1 week
3. A pyramid of resources<br>*[Overview of the architecture of evidence]*	* When clicking on the link under the pyramid: "6S model explained"	After 2 weeks
4. Is one summary enough?<br>*[Top layers: Summaries]*	* After 2 searches when only PubMed links are clicked (regardless of whether filtered or unfiltered)<br>* After 2 searches when only the same summary is clicked (if this is too specific to be implemented, change to when 3 summaries are clicked.	After 3 weeks
5. New and critically appraised evidence<br>*[Middle layers: Preappraised research]*	* After 4 searches when clicking on PubMed only (filtered or unfiltered)	After 4 weeks
6. PubMed & the Clinical Queries<br>*[Bottom layers: Non-preappraised research]*	*After 6 searches clicking only on unfiltered PubMed (if this is too specific to be implemented, change to after 6 searches clicking only on PubMed)	After 5 weeks
7. Preparing searchable questions<br>*[Using the PICO framework]*	* After 2 searches without clicking on any citation.<br>*After 2 searches with no citations retrieved in middle layers (i.e. excluding summaries and PubMed)	After 7 weeks
8. Academic work<br>*[Using a federated search for presentations, grants and research]*	*When trying to download citations	After 8 weeks
[Any video]	Then display random video	Weekly until the end of the trial
