# Supplementary material for: Increasing the quantity and quality of searching for current best evidence to answer clinical questions: protocol and intervention design of the MacPLUS FS Factorial Randomized Controlled Trials
Source: Implement Sci. 2014 Sep 20;9:125. doi: 10.1186/s13012-014-0125-9 (PMC4177052; doi:10.1186/s13012-014-0125-9)
Supplement: Supplementary file 3 — Additional file 3: Online administration of Impact Assessment Method (IAM) questionnaire.(PDF 191 KB) [file 13012_2014_125_MOESM3_ESM.pdf]

# <u>Additional file 3</u>: Online administration of Impact Assessment Method (IAM questionnaire)

---

- The first invitation will be sent after the first search, one month following the first exposure to the intervention(s), i.e. one month after the participant had any interaction with MacPLUS FS.
- If no answer, one reminder will be sent at 24 hours.
- Once a questionnaire is filled (qualifies as filled if Q1+Q2+Q3 are answered), no further questionnaire is sent.
- If not, another survey will be sent following the next search after a 2 weeks delay, until a filled questionnaire is returned, or the trial ends.
- IAM is adapted from:
  - Grad R, Pluye P, Granikov V, Johnson-Lafleur J, Shulha M, Sridhar, S. B.: **Physicians' assessment of the value of clinical information: Operationalization of a theoretical model.** *Journal of the American Society for Information Science and Technology* 2011, 62:1884-1891.
  - Pluye P, Grad RM, Granikov V, Jagosh J, Leung K: **Evaluation of email alerts in practice: part 1 - review of the literature on clinical emailing channels**. *J Eval Clin Pract* 2010, 16:1227-1235.
  - Pluye P, Grad RM, Johnson-Lafleur J, Bambrick T, Burnand B, Mercer J, Marlow B, Campbell C: **Evaluation of email alerts in practice: Part 2 - validation of the information assessment method.** *J Eval Clin Pract* 2010, 16:1236-1243.

---

# A. Invitation e-mail

MacPLUS Federated Search — Evidence Based

Dear Doctor [Agoritsas],

On [date] at about [time] you searched for "[search string]" in MacPLUS FS.

We would like to ask a few quick questions about that search. This brief survey includes 1 to 6 questions that would take < 1 minute of your time to answer:

- Yes I agree to participate to the brief survey
- No thanks

We appreciate your help in improving access to current best evidence through MacPLUS FS.

Thank you,

R. Brian Haynes, MD, PhD, FRCPC, FRSC
Chief, Health Information Research Unit (http://hiru.mcmaster.ca)
Faculty of Health Science

# B. IAM Survey

Dear Doctor Agoritsas,

On [date] at about [time] you searched for "[searchstring]" in MacPLUS FS.

**Q1. Do the term(s) in this search represent an attempt you made to answer a question of relevance to your clinical interests?**

- Yes
- No

**Q2. Why did you do this search for information?** ***Please check all that apply.*** *Note: You can check more than one objective.*

- To address a clinical question (problem) about a specific patient
- To fulfill a personal educational objective
- To satisfy curiosity or for personal interest
- To look up something I had forgotten
- To share information with a patient, their family, or home health aides
- To exchange information with other health professionals (e.g., a colleague)
- To manage aspects of patient care with other health professionals

**Q3. Did you find relevant information that partially or completely met your objective(s)?**

- Yes
- No

**Q4. What is the impact of this information on you or your practice?** ***Please check all that apply.*** *Note: You can check more than one type of impact.*

- My practice was (will be) changed and improved
- I learned something new
- This information confirmed I did (am doing) the right thing
- I am reassured

- [ ] I am reminded of something I already knew
- [ ] I am dissatisfied
- [ ] There is a problem with the presentation of this information
- [ ] I disagree with the content of this information
- [ ] This information is potentially harmful

**Q5. Did you (will you) use this information for a specific patient?**

○ Yes ○ No ○ Possibly

***Please check all that apply.*** *Note: You can check more than one type of use.*

- [ ] As a result of this information I managed (or will manage) this patient differently
- [ ] I had several options for this patient, and I used (will use) this information to justify a choice
- [ ] I did not know what to do, and I used (will use) this information to manage this patient
- [ ] I thought I knew what to do, and I used this information to be more certain about the management of this patient
- [ ] I used this information to better understand a particular issue related to this patient
- [ ] I used (will use) this information in a discussion with this patient, or with other health professionals about this patient
- [ ] I used (will use) this information to persuade this patient, or to persuade other health professionals to make a change for this patient

*(if no or possibly, skip to Q7)*

**Q6. For this patient, did you observe (or do you expect) any health benefits as a result of applying this information?**

○ Yes ○ No ◉ Possibly

***Please check all that apply.*** *Note: You can check more than one type of health benefit.*

- [ ] This information helped to improve (will help to improve) this patient's health status, functioning or resilience (i.e., ability to adapt to significant life stressors)
- [ ] This information helped to prevent (will help to prevent) a disease or worsening of disease for this patient
- [ ] This information helped to avoid (will help to avoid) unnecessary or inappropriate treatment, diagnostic procedures, preventative interventions or a referral, for this patient
- [ ] This information helped to decrease this patient's worries about a treatment, diagnostic procedure or preventative intervention
- [ ] This information helped to increase this patient's knowledge, or their family or home health aides' knowledge

## C. Note after answering “no” to the invitation e-mail

Want to do a search in MacPLUS FS? click here

Thank you for your answer. We understand that you were not able answer our brief survey this time. As we continuously aim to improve the clinical usefulness of MacPLUS FS we may invite you to a future survey in a few weeks.

Thank you in advance for considering,

R. Brian Haynes, MD, PhD, FRCPC, FRSC
Chief, Health Information Research Unit (http://hiru.mcmaster.ca)
Faculty of Health Science

## D. Note after answering “no” to Q1

Want to do a search in MacPLUS FS? click here

Thank you for your answer. As we continuously aim to improve the clinical usefulness of MacPLUS FS we may invite you to a future survey in a few weeks.

Thank you in advance for considering,

R. Brian Haynes, MD, PhD, FRCPC, FRSC
Chief, Health Information Research Unit (http://hiru.mcmaster.ca)
Faculty of Health Science
