# Supplementary material for: Increasing the quantity and quality of searching for current best evidence to answer clinical questions: protocol and intervention design of the MacPLUS FS Factorial Randomized Controlled Trials
Source: Implement Sci. 2014 Sep 20;9:125. doi: 10.1186/s13012-014-0125-9 (PMC4177052; doi:10.1186/s13012-014-0125-9)
Supplement: Supplementary file 4 — Additional file 4: Power curves for the primary outcome.(PDF 48 KB) [file 13012_2014_125_MOESM4_ESM.pdf]

<u>Additional File 4</u>. Power as a function of difference in means, separately for our samples of postgraduates (n=429) and faculty (n=475)

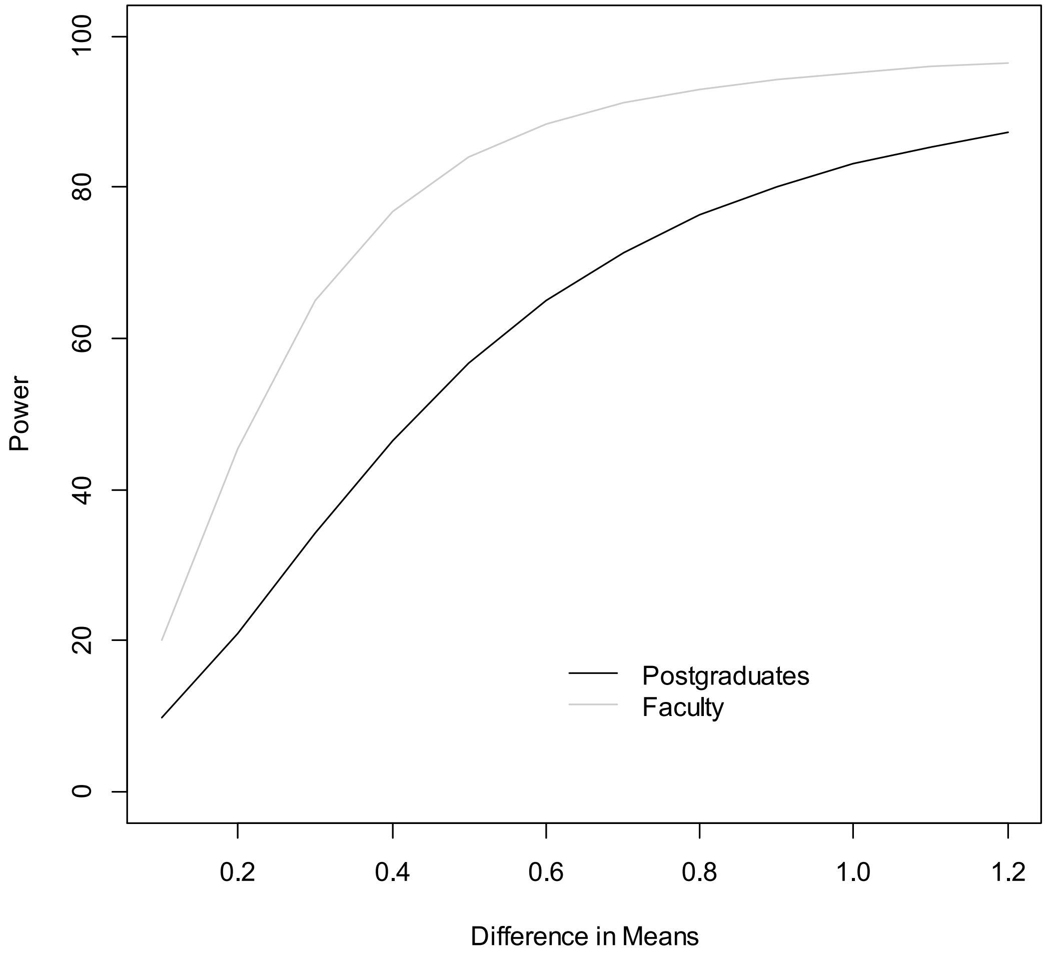

The distribution of searches is such that the standard deviation varies with the mean; baseline data suggested a linear relationship, with the standard deviation of searches in any given month being approximately 0.15 + 4.84*mean. Since the primary analysis will use the mean number of searches per individual over a 6-month time frame, the actual standard deviation will be smaller than this; the standard deviation in this case is made up of within-individual variability and between-individual variability, and averaging over several months will diminish the within-individual variability. Baseline data suggested that taking 6-month averages reduced the standard deviation by 30%, and therefore the standard deviation was modelled as (0.15 + 4.84*mean)*0.7. Power calculations use a two-sample t-test for unequal variances. The planned analysis will use regression to adjust for stratifying baseline variables, making this analysis slightly conservative.
