# Supplementary figures and images for: Increasing the quantity and quality of searching for current best evidence to answer clinical questions: protocol and intervention design of the MacPLUS FS Factorial Randomized Controlled Trials
Source: Implement Sci. 2014 Sep 20;9:125. doi: 10.1186/s13012-014-0125-9 (PMC4177052; doi:10.1186/s13012-014-0125-9)

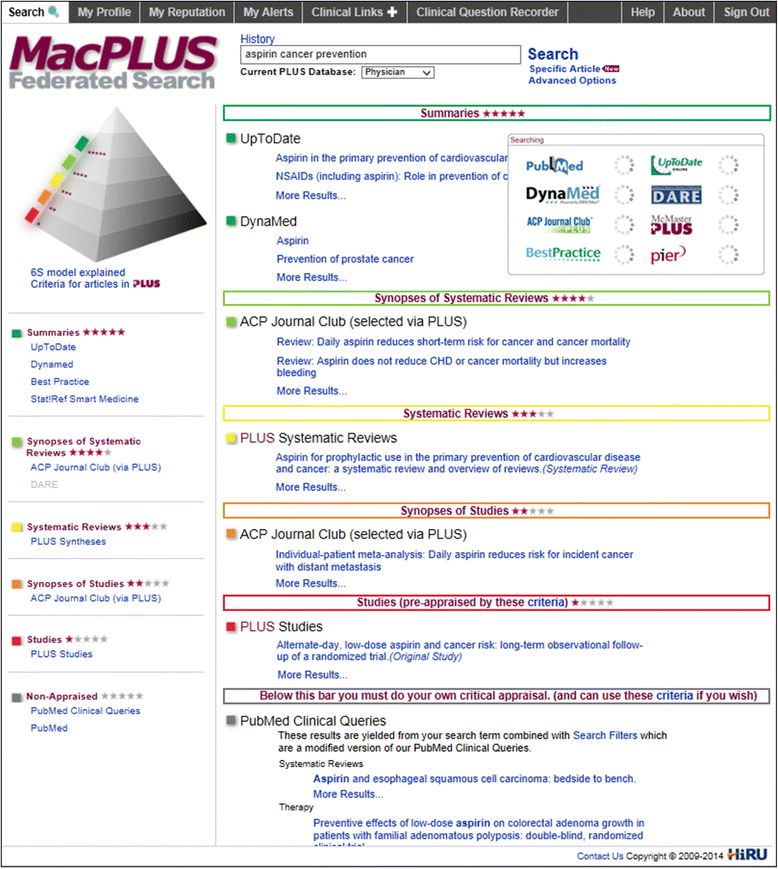

Supplement: Supplementary file 5 — Authors’ original file for figure 1 [file 13012_2014_125_MOESM5_ESM.gif]

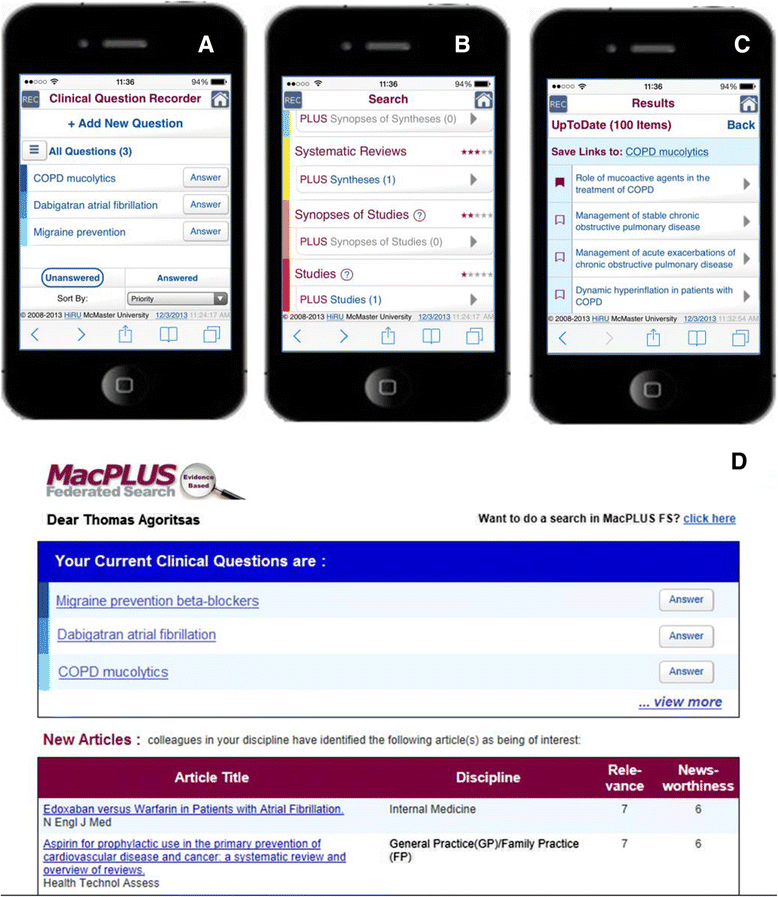

Supplement: Supplementary file 6 — Authors’ original file for figure 2 [file 13012_2014_125_MOESM6_ESM.gif]

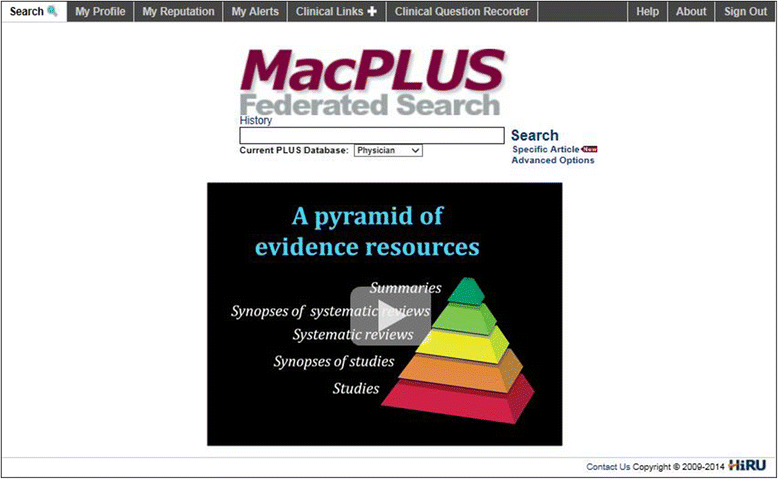

Supplement: Supplementary file 7 — Authors’ original file for figure 3 [file 13012_2014_125_MOESM7_ESM.gif]

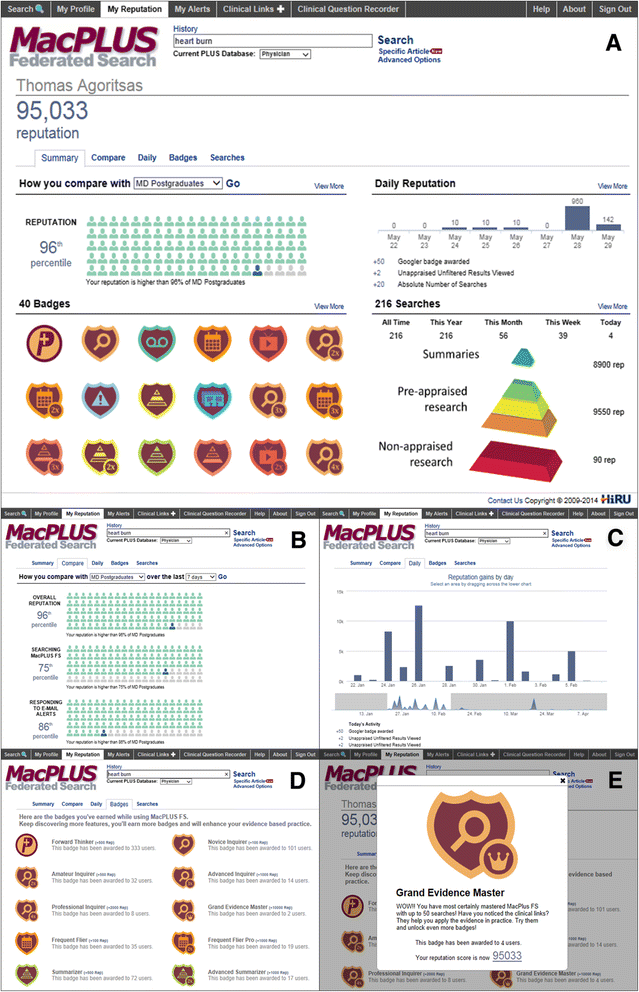

Supplement: Supplementary file 8 — Authors’ original file for figure 4 [file 13012_2014_125_MOESM8_ESM.gif]
